# The Application of Electricity in Medicine and Surgery*Presented to the meeting of the Alumni, July 22, 1898.

**Published:** 1898-07

**Authors:** J. McFadden Gaston

**Affiliations:** Atlanta, Ga.; President Alumni Association of Southern Medical College


### Southern Medical Record.

A MONTHLY JOURNAL OF MEDICINE AND SURGERY.

VOL. XXVIII. ATLANTA, GA., JULY, 1898. NO. 7.

### Original Articles.

### THE APPLICATION OF ELECTRICITY IN MEDICINE AND SURGERY.*

By J. McFADDEN GASTON, Jr., A.M., M. D., Atlanta, Ga.

President Alumni Association of Southern Medical College.

Electricity has been applied in all the arts and sciences and to a great variety of useful mechanical appliances. The thorough realization of the scope of this wonderful agent is not possible without considering and contemplating the many changes which have been brought about since the important discovery by Galvani.

The electric telegraph first startled the world by the rapidity of transmission and the annihilation of time and space. Then the telephone became a complete revelation of power, and a new impetus to all communication between man and man was given when its many improvements were applied.

In like manner as a motor, electricity has made changes in locomotion in every department of enterprise.

While the changes have been more wide-spread in the above applications of electricity, we doubt whether there could be a more astonishing transformation of methods than that accomplished by the application of electricity to medicine and surgery. Beginning with the Leyden jar, a simple shock was used in certain diseases, while the static machine has been known for centuries to be a powerful therapeutic remedy.

At the same time we find that the most important results have been attained by Galvanic and Faradic electricity.

In treating strictures, tumors, naevi, nervous diseases, the current has been applied with marked and satisfactory cures. The careful observation of data and the compilation of statistics by Apostoli, Betton Massey, and others show that in diseases of women electrolysis is an agent of undoubted merit. In the method of cataphoresis as used by Drs. McGuire, Gaston and others, most remarkable changes in cell formation and

*Presented to the meeting of the Alumni, July 22, 1898.

tissue proliferation are shown to be made by medicines which can be directly topically applied by the method of transmission from the positive to the negative pole. The ordinary stimulant effect of electricity enables the physician to administer medicines by its means which would not be borne so well by weak patients. Carcino-sarcoma, Adeno-sarcoma, or small celled sarcoma have been cured by the application of Donovan's solution to them in this way. At the same time an internal exhibition of this drug has been used.

The process of the galvano-cautery has been very well recognized, and by means of snares nasal polypi and adenoid growths have been successfully extirpated, while the hemorrhage in these cases is less than in the process of torsion or of the various cutting instruments that have been devised.

In regard to the cauterizing effects of electricity, we would mention Dr. Betton Massey's process of cataphoresis and the action of gold electrodes coated with pure mercury. The cases which he has treated are cases of malignant growths which are frequently operable, but which in his experience have yielded to the destructive effects of the mercury and the current combined. The cases which he has reported recently have been cases of carcinoma of the mammary gland, and in these cases the axillary glands are also attacked by the electrode in one seanc. under an anæsthetic.

Dr. E. H. Bradley, of Peoria, Illinois, in a thoughtful article in the Peoria *Medical Journal* of July, 1898, draws attention to the value of cataphoresis in the sterilization of wounds, showing the direct application of mercury when the bichloride is used and the free chlorine escapes at the positive pole where the bichloride of mercury is applied, while the metallic mercury goes to the interior of the wound and to the negative pole, killing all germs.

He states that iodine is electro-positive and hence must be applied to the negative pole of the current, but the results of McGuire in goitre do not bear him out.

The application of the weak galvanic current has also been successfully made in cases of urethral stricture, enlarged prostate; and epilation by electrolysis is a very well recognized process.

I have recently treated a case by electricity in a rather novel condition. The nerve of the arm was painful and a small amount, about one-half grain of cocaine hydrochlorate was applied to the arm along the ramifications of the nerve, and this is always attended with anæsthesia as is known in cases of extraction of teeth. Another case now under my care is one of a lady with a few extra hairs on both sides of upper lip, and several on the chin and the neck. The needle is put on the negative pole and applied into the hair follicle. Half a minute suffices to destroy the follicle. About five cells of a Waite and Bartlett battery are used.

Case of a uterine fibroid was seen this week, with the advice to have electricity applied.

